# The Relationship between Baseline Neutrophils Counts and Response to 12 Weeks of Antipsychotics in First-Episode Psychosis

**DOI:** 10.21203/rs.3.rs-8502917/v1

**Published:** 2026-01-20

**Authors:** Gauri Shastri, Jose Rubio, Juan Gallego, Delbert Robinson, Anil Maholtra, Todd Lencz

**Affiliations:** Northwell Health; Northwell; The Zucker Hillside Hospital; Zucker Hillside Hospital; Northwell Health

## Abstract

Multiple pathways of immune system activation have been implicated in psychosis. Elevated neutrophils and neutrophil-lymphocyte ratio (NLR) as a marker of inflammatory response have been well studied in case-control studies of schizophrenia and first-episode psychosis. However, only one prior study has examined the potential of baseline neutrophil counts or NLR to predict treatment outcomes in first-episode psychosis. We examined whether absolute and relative neutrophil counts at baseline could predict positive symptom improvement in a cohort of 85 patients with first-episode psychosis who received up to 12 weeks of antipsychotic treatment. We used logistic regression with treatment response (defined as the absence of psychotic-level positive symptoms) as the binary outcome of interest and absolute/relative neutrophil measures as the predictor variables of interest, controlling for the following covariates: smoking status, BMI, antipsychotic naïve status, race, and sex. We found that higher NLR (OR 0.53, 95% CI 0.28–0.96, p = 0.041) and neutrophil relative count (OR 0.91, 95% CI 0.84–0.98, p = 0.013), but not absolute counts (OR 0.86, 95% CI 0.6–1.23, p = 0.39), were significantly associated with decreased likelihood of treatment response. We have thus demonstrated that baseline NLR is inversely correlated with antipsychotic treatment response in the acute phase of treatment for patients with first-episode psychosis. Although effect sizes were too small for individual-level prediction, our findings suggest a potential role for inflammatory markers in predicting treatment response. Further studies are needed to identify more specific mechanisms underlying this association.

## Introduction

Multiple pathways of immune dysregulation have been implicated in psychotic spectrum illness^1^. While many approaches to measuring immune system activation and/or inflammatory response have been proposed, one readily ascertained measure with considerable support in the literature is the assessment of neutrophils^2-5^. A recent meta-analysis of case-control studies has demonstrated innate immune system activation as measured by elevated absolute neutrophil counts, neutrophil percentage, and/or neutrophil-lymphocyte ratio (NLR) in schizophrenia broadly, but especially in first-episode psychosis (FEP)^2^. Other studies have prospectively examined changes in neutrophil counts over time in response to antipsychotic treatment^3,6-8^, and studies have also demonstrated a positive correlation between neutrophil counts or NLR with acute psychosis severity^3,9^. However, only one study to our knowledge has examined baseline neutrophil counts as a direct indicator of antipsychotic treatment response in FEP^10^. In this study, we address this gap in the literature by examining the relationship between neutrophil counts (absolute and relative) and NLR with acute positive symptom response in a first-episode psychosis cohort within the first 12 weeks of antipsychotic treatment. We focused on positive symptoms of psychosis since the currently available antipsychotic medications most effectively target these symptoms^11^. We also incorporate cigarette smoking status and body mass index (BMI) information in our study, as both these measures are well-known influencers of inflammation as measured by neutrophil levels, though how this relates to neutrophil elevation in psychosis remains an open question^2,3,12^.

## Methods

### Participant Recruitment and Diagnostic Assessment

The participants of this study were individuals aged 16–40 years with affective and non-affective first-episode psychosis and no more than 1 month of total prior exposure to antipsychotics at study entry. These individuals were recruited and evaluated at Zucker Hillside Hospital with methods consistent with prior studies at this site^13^. Briefly, initial diagnosis was determined by Structured Clinical Interview for DSM-IV Axis I Disorders (SCID) and confirmed by consensus evaluation. All study participants provided written informed consent, and data collection was approved by the Feinstein Institutes for Medical Research Institutional Review Board (IRB). All study participants also had baseline peripheral blood collected and processed at a Northwell Health Core Lab using the Sysmex^™^ hematology analyzer, which relies on impedance technology and flow cytometry to determine absolute and relative blood counts (Sysmex Corporation, Kobe, Japan).

### Antipsychotic Treatment and Response Assessment

Recruited patients received up to 12 weeks of prospective antipsychotic treatment with either aripiprazole or risperidone, given that our prior work demonstrated no difference in positive symptom response or overall response times between these two medications^13^. Treatment response status was evaluated weekly for the first 4 weeks, then every two weeks through week 12. As described in our prior publications^13^, treatment response was defined as two consecutive ratings of 1 (“very much improved”) or 2 (“much improved”) on the CGI-I, as well as a rating of less than or equal to 3 on four psychosis-related items of the BPRS-A. Individuals who met these criteria at any point during the 12 week trial were classified as responders. All others participants were classified as non-responders, as long as they remained in the study for at least four weeks. Those who dropped out within 4 weeks of study entry without demonstrating response were excluded, because they did not receive an adequate trial of antipsychotic treatment.

### Data Analysis

We first examined the bivariate relationship between BMI or smoking status and three neutrophil-related outcome variables of interest. We then performed a logistic regression with treatment response status as the binary outcome variable and absolute/relative neutrophil counts as the predictor variable of interest. Because prior literature in this area has utilized varying measures to capture neutrophil levels^2,4^, we performed separate regressions using neutrophil percentage, neutrophil count, or NLR as the predictor. Each model included the following covariates: biological sex, race (Black, White, or Other), baseline antipsychotic naïve status, baseline smoking status, and baseline BMI prior to antipsychotic treatment.

## Results

Of the recruited subjects with available treatment response data (*n = 121*), we excluded 26 individuals who dropped out within 4 weeks of study entry without meeting response criteria, because they did not receive an adequate trial of antipsychotic treatment. These early dropouts did not differ significantly in age, race, and antipsychotic naïve status compared to study subjects, but were more likely to be women (*p = 0.037*).We also excluded individuals with missing blood data , individuals with more than 15 days elapsed between date of study entry and baseline blood draw, and one individual with a baseline lymphocyte count greater than 20,000/uL (more than 10 standard deviations above the mean), resulting in *n = 8*5 subjects to be included for further analysis (see **Table 1** for demographic characteristics). Nearly half of the patients (37 of the 85 subjects, 44%) were antipsychotic naïve at baseline, and among the 48 subjects with prior antipsychotic exposure, 42 subjects had available the number of days of prior exposure. The mean and median number of days of antipsychotic exposure prior to study entry was 4.05 days and 1 day, respectively.

**Figure 1:**
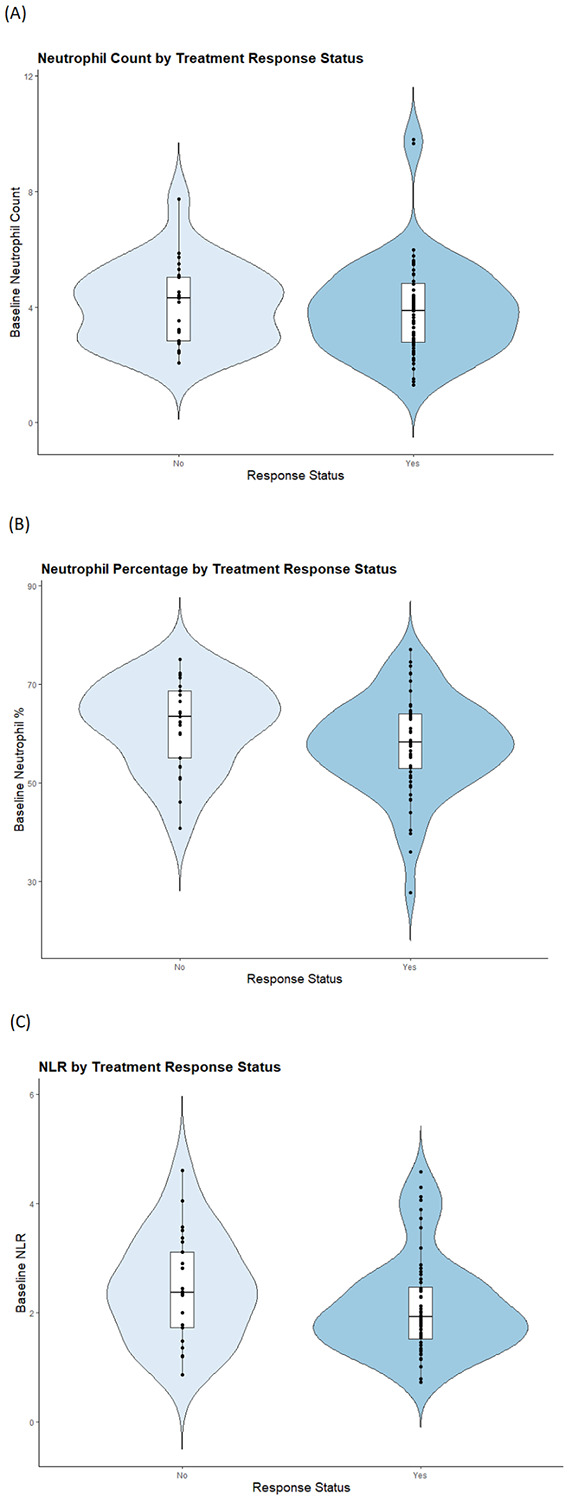
The relationship between neutrophils and treatment response status in first-episode psychosis: Violin plot with embedded boxplot demonstrating the difference between baseline neutrophil count (A), neutrophil percentage (B), or NLR (C) between treatment responders versus non-responders after up to 12 weeks of antipsychotic treatment.

We first examined the bivariate relationships between baseline BMI or baseline smoking status and the three outcome variables of interest (absolute neutrophil count, relative neutrophil count, and NLR). We found that absolute neutrophil count was significantly correlated with baseline BMI (*p = 0.001*) and NLR significantly differed between baseline smokers and non-smokers (*p = 0.009*). Thus, both smoking status and BMI were included in our regression models as potential influencers of neutrophil levels. Results of logistic regression analysis demonstrated that higher NLR (*OR = 0.53, 95% CI = 0.28 – 0.96, p = 0.041*, see Figure 1) and higher relative neutrophil count (*OR = 0.91, 95% CI = 0.84 – 0.98, p = 0.013*) were significantly associated with greater likelihood of treatment nonresponse. There was no significant relationship between treatment response and absolute neutrophil count (*OR = 0.86, 95% CI = 0.60 – 1.23, p = 0.39*). In all three models, treatment response was not significantly associated with race, sex, or baseline BMI, smoking status, or antipsychotic naïve status. Mean absolute neutrophil count was significantly lower in Black study subjects compared to all other subjects (*p = 0.011*), as expected based on prior literature on the Duffy-null phenotype contributing to clinically-benign lower absolute neutrophil counts (ANC) in many people of African ancestry^14^. However, results remained unchanged when the same models were run with Black race status as a binary covariate.

## Discussion

While there is replicated evidence that first-episode psychosis is associated with elevated neutrophil counts and NLR^2-4,6-10^, only one study to-date has examined white blood cell composition as a direct prognostic measure for antipsychotic treatment response^10^. In the present study, we found an inverse relationship between NLR at baseline and positive symptom response to antipsychotic after 12 weeks of treatment in first-episode psychosis patients. This relationship was also true for relative neutrophil count at baseline, although there was no significant association between absolute neutrophil count and treatment response. These findings were independent of race, biological sex, baseline BMI and smoking status, and whether the patients were antipsychotic-naïve at baseline, though median prior antipsychotic exposure was less than 1 week.

This finding is in direct contrast with the work by Lu *et al*^10^, which demonstrated a positive correlation between both neutrophil count and NLR with symptom improvement after 12 weeks of monotherapy with risperidone in medication-naïve patients with “early schizophrenia”. This difference may be in part due to two major differences in diagnostic inclusion criteria: 1) our study examined acute response to the first medication trial, whereas the study of Lu *et al*^10^ included patients within five years of illness onset; and 2) our study was more broadly inclusive of first-episode psychosis including affective illness, as patients are often diagnostically undifferentiated in their index episode of illness. Our study cohort also captured a marginally younger population, which may impact disease prognosis. For example, a cross-sectional study by Wang *et al.*^15^ found that NLR and neutrophil count were positively correlated with PANSS scores in antipsychotic-naïve first-episode patients with early-onset schizophrenia but not those with onset after age 21; however, this finding may be related to differences in disease severity as the early-onset group had significantly worse negative symptoms as measured on PANSS.

Our demonstrated association between baseline neutrophil measures and treatment outcome is consistent with the longitudinal retrospective study by Llorca-Bofi *et al.*^16^, which found that elevation in multiple leukocyte measures including neutrophils and NLR at time of schizophrenia spectrum diagnosis was associated with later clozapine prescription or ECT, both markers of treatment resistance. Furthermore, multiple studies have prospectively monitored blood leukocyte measures over the course of antipsychotic treatment^2,3,6-8,17,18^, and Steiner *et al.*^3^ found significantly lower neutrophil count and NLR after 6 weeks of antipsychotic treatment in both FEP and chronic schizophrenia patients, though not to the level of healthy controls. Notably, the reduction of neutrophil count correlated with the improvement of positive symptoms as measured by PANSS-P^3^. Similarly, Bioque *et al.*^17^ found that the FEP patients who did not achieve remission after two years had a significantly higher NLR at that time. These observations are consistent with our finding that lower neutrophil levels suggest better prognosis and support studies on other inflammatory marker changes in early psychosis. For example, Mondelli *et al.*^19^ demonstrated that lower baseline levels of complement C4 was associated with treatment response after 1 year in first-episode psychosis. More broadly, Korhonen *et al.*^20^ found multiple plasma proteins related to the acute phase immune response, including the neutrophil-derived EN-RAGE, to be elevated in antipsychotic-naïve FEP patients compared to healthy controls.

Our results complement existing studies on inflammatory changes in chronic schizophrenia including later illness exacerbation. For example, in patients with an established diagnosis of schizophrenia who were hospitalized for an acute exacerbation of symptoms, Labonté *et al.*^7^ found NLR to be elevated at time of discharge in treatment-resistant patients compared to treatment-responsive patients, despite NLR being comparable on admission. Özdin and Böke^21^ also found NLR to be elevated during symptomatic relapse in patients with schizophrenia. Thus, baseline differences in inflammatory markers may become more pronounced over the course of the disease.

The mechanistic link between innate immune system activation and psychosis remains unknown^2,22^, yet dysregulation of a broad array of immune system markers have been linked to schizophrenia, including elevation of cytokines such as IL-6, maternal immune activation during the prenatal period, complement C4 abnormalities, and increased microglial activity ^1,23^. Given that chronic inflammation is associated with neuronal damage and cognitive decline^1^, perhaps nonspecific immune overactivity both predisposes an individual to psychosis and exacerbates illness severity.

There are several limitations of the present study. First, prior studies have suggested that timing of blood draws may impact circulating immune cell counts, though a schizophrenia case-control study by Villar *et al.*^24^ found that differences in neutrophil count and NLR remained significant when accounting for the specific time of blood draw as well as the subject’s medication status. In the present study, labs were typically drawn in the morning, but time of collection was not explicitly recorded. Second, early study dropouts who were non-responders were more likely to be women, though other demographic characteristics remained comparable, and biological sex was not significantly associated with treatment response in our study cohort of 85 subjects. Third, the first-episode psychosis cohort of this study included both primary psychosis and affective psychosis diagnoses, as is often the case in early psychosis studies. However, the study by Bioque *et al.* did not find any significant difference in NLR between affective and non-affective psychosis at baseline or at two-year follow-up^17^, suggesting that blood leukocyte measures may not be significantly impacted by diagnostic categorization. Diagnostic differences may become more prominent when considering more expansive biomarkers and/or later disease stages: for example, one study developed a machine-learning model integrating blood-based immunological biomarkers and cognitive data to predict an established diagnosis of bipolar disorder versus schizophrenia^25^. Finally, it remains an open questions how substance use and metabolic syndrome may mediate inflammation observed in patients with schizophrenia^2^, though incidence of smoking at baseline was lower in this study (14%, see Table 1) compared to historic levels in the FEP population. While case-control studies report increased NLR in substance use disorders compared to healthy controls, there is evidence that NLR may be higher in primary psychosis compared to substance-induced psychosis^26^.

In conclusion, our study is one of the first to demonstrate a potential prognostic role for neutrophil levels to predict response to antipsychotics in first-episode psychosis. This finding complements existing evidence that immune markers such as neutrophil count and NLR may mirror treatment response trajectories and illness exacerbation when monitored over time. Future studies should incorporate more expansive metabolic and inflammatory markers to better elucidate the relationship between neutrophils and psychosis treatment response to determine opportunities for impactful clinical intervention.

## Supplementary Material

This is a list of supplementary files associated with this preprint. Click to download.

• NeutrophilsFEPTable.docx
